# Hands-Free Human-Computer Interface Based on Facial Myoelectric Pattern Recognition

**DOI:** 10.3389/fneur.2019.00444

**Published:** 2019-04-30

**Authors:** Zhiyuan Lu, Ping Zhou

**Affiliations:** Department of Physical Medicine and Rehabilitation, University of Texas Health Science Center at Houston, and TIRR Memorial Hermann Research Center, Houston, TX, United States

**Keywords:** human-machine interface, hands-free interaction, surface EMG, facial motion recognition, pattern recognition

## Abstract

Patients with no or limited hand function usually have difficulty in using conventional input devices such as a mouse or a touch screen. Having the ability of manipulating electronic devices can give patients full access to the digital world, thereby increasing their independence and confidence, and enriching their lives. In this study, a hands-free human-computer interface was developed in order to help patients manipulate computers using facial movements. Five facial movement patterns were detected by four electromyography (EMG) sensors, and classified using myoelectric pattern recognition algorithms. Facial movement patterns were mapped to cursor actions including movements in different directions and click. A typing task and a drawing task were designed in order to assess the interaction performance of the interface in daily use. Ten able-bodied subjects participated in the experiment. In the typing task, the median path efficiency was 80.4%, and the median input rate was 5.9 letters per minute. In the drawing task, the median time to accomplish was 239.9 s. Moreover, all the subjects achieved high classification accuracy (median: 98.0%). The interface driven by facial EMG achieved high performance, and will be assessed on patients with limited hand functions in the future.

## Introduction

Human facial movements have been established to be reliable input to a variety of machines including electronic devices (1–6) and assistive devices (7, 8). Such kind of human-machine interface provides an easy and intuitive approach to interacting with electronic devices for users with limited hand function (9). As a result, they have improved accessibility to the digital world and better user experience.

Facial movements can be detected by techniques such as electrophysiological and kinematic recordings (5–7). Surface electromyography (EMG) is one of the electrophysiological techniques. It records muscle activity using electrodes that are usually attached to the skin in various body locations. EMG can be used to detect movement (10–12), and its robustness in the application of human-machine interaction has been demonstrated in recent studies (13–15). The conventional mapping scheme between facial movements detected by EMG signals and control commands involves channel-based mapping. Often, one EMG channel is mapped to one control command, or a group of EMG channels (typically two channels, one from an agonist muscle and the other from an antagonistic muscle) are mapped to a group of control commands (usually two opposite commands). For example, Williams et al. (1) recorded four EMG channels, and mapped them to four different commands. Similarly, Cler et al. (2) mapped five EMG channels to five control commands. In their design, opposite commands such as moving the cursor up or down were generated from two EMG channels recorded from two facial muscles. For example, the cursor moved upward if the EMG from the Frontalis was larger than from the Mentalis, and downward if it was smaller. Such a conventional mapping scheme requires the number of EMG channels to be the same as the number of control commands, and each channel needs to be placed on one muscle or muscle group that conducts the movement. However, due to the limited degrees of freedom of facial movements, it is challenging to find a large number of muscles that can be individually activated. Moreover, EMG amplitude or amplitude range of different muscles can be different, even at the same force level. As a result, each channel has to be individually calibrated (i.e., threshold, gain, etc.) (2).

To address these challenges, a pattern-based mapping scheme was introduced. Such a scheme relies on myoelectric pattern recognition, which is based on the assumption that each movement pattern has a unique muscle activation pattern that can be recorded by EMG. Different from conventional mapping, a pattern-based scheme classifies movement patterns by applying pattern recognition algorithms to comprehensively analyze all EMG channels, and maps each movement pattern to a control command. Pattern recognition algorithms can learn the user's movement patterns, so that there is no need to calibrate each channel manually. In addition, both single-muscle movements and complex movements (e.g., motions that are conducted by temporal or spatial coordination of multiple muscles) can be mapped by the algorithms as movement patterns. Thus, the number of EMG channels can be fewer than the number of control commands. This mapping scheme has been widely applied in myoelectric human-machine interfaces (14), especially when hand movements are mapped to control commands (16–18). Although some recent studies have demonstrated the feasibility of hand pattern recognition in amputees (19) and patients with limited hand function (20, 21), there is still a large patient population whose hand movement patterns cannot be well detected or classified using limb EMG (22, 23). A human-machine interface based on facial movements is particularly useful for these patients to gain the ability of manipulating electronic devices. Previous studies have shown that it is possible to detect and classify facial movements at a high accuracy using only a few EMG channels (24–26). However, few studies have investigated a control scheme driven by myoelectric pattern recognition of facial movements (3, 27), and its performance still remains unclear.

This study aims to develop a facial movement-machine interface (FMMI) that maps facial movements to cursor actions including cursor movements in different directions and cursor click, so that users with no or limited hand function can use computers or other computer-controlled devices. To evaluate the performance of the developed FMMI, the accuracy of the real-time myoelectric facial movement recognition was determined. The interaction performance based on the recognition results was also measured in a typing task and a drawing task.

## Methods

### System Architecture

**Figure 1:**
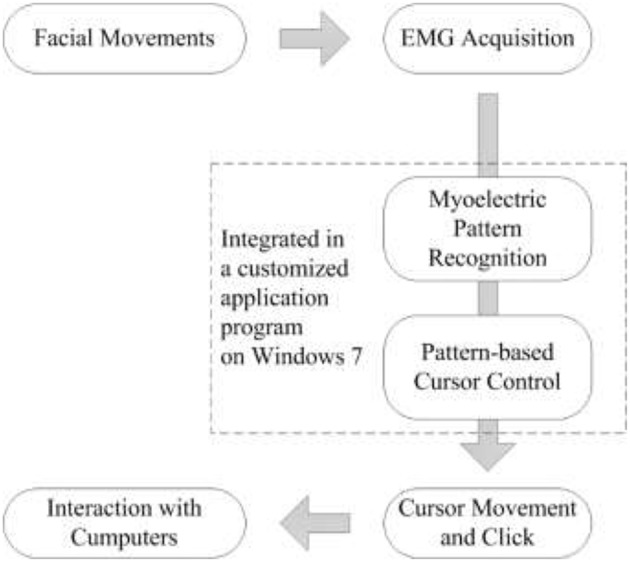
Architecture of the facial movement-machine interface (FMMI).

The FMMI consists of EMG acquisition devices and a personal computer that the user manipulates (Figure 1). A customized computer program detects and classifies the user's facial movements using integrated pattern recognition algorithms, and controls the system cursor to drive computer operations.

### Interaction Design

**Figure 2:**
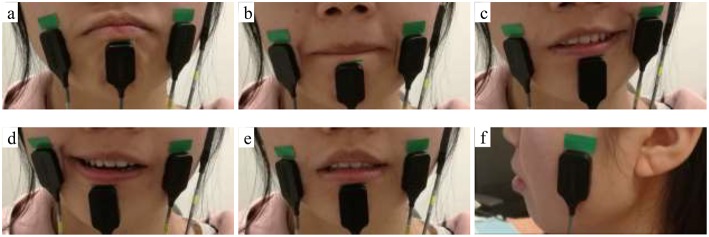
Facial movements **(a)** Raise lower lip, **(b)** Close lips, **(c)** Left side smirk, **(d)** Right side smirk, **(e)** Bite down; electrodes were placed on Left & right Risorius, Mentalis, and Temporalis muscles **(a–f)**.

The FMMI aims to provide users with the same experience as using a regular mouse. Therefore, continuous mapping (2) is applied in the design. In other words, the cursor moves when the user's facial muscles are activated, and vice versa. Five facial movement patterns were defined (Figure 2) and mapped to five cursor actions; cursor movements in four different directions and left button click (Table 1). In order to accept the drag-and-drop operation, a “long” bite-down motion, which lasts for more than 1.5 s, was mapped to drag (i.e., left button press and hold). A “short” bite-down motion, which lasts for no more than 1.5s was mapped to either left button release or click (left button press and then release). As a result, the user can drag an object using a “long” bite-down motion, and then move it anywhere using only left/right Risorius or Mentalis muscles, and drop it by doing a “short” bite-down.

**Table 1:** Mapping between Facial Movements and Cursor Actions.

**Facial movement**	**Cursor action**	**Muscle**
Left side smirk	Move left	Left risorius
Right side smirk	Move right	Right risorius
Raise lower lip	Move up	Mentalis
Close lips	Move down	Left & right risorius, mentalis
Bite down	Left button ^a^	Temporalis
No movement	No action	None

a *A “long” bite-down motion that lasts for more than 1.5s is mapped to drag (left button press and hold). A “short” bite-down motion that lasts for no more than 1.5s is mapped to either left button release or click (left button press and then release)*.

### EMG Acquisition

Four EMG channels were recorded, from Mentalis, left Risorius, right Risorius, and left Temporalis muscles. The skin was first cleaned using alcohol wipes, and EMG sensors (Delsys 2.1 single differential configuration, Delsys Inc., Boston, USA) were placed over the muscles using double-sided adhesive tape. The reference electrode was placed on right clavicle. The raw EMG signals were filtered (20–450 Hz band pass filter) and amplified 10,000 times by a Delsys Bagnoli-8 EMG System (Delsys Inc., Boston, USA). The EMG signals were digitized at a sampling rate of 1,000 Hz by a 16-bit analog-to-digital converter (USB-6221, National Instruments Inc., Austin, USA), recorded on a computer (Windows 7, Intel Celeron 1007 U at 1.5 GHz with 4 GB RAM) via USB, and accessed using a National Instruments programming interface (NI-DAQmx) for C++.

### EMG Signal Processing

#### Segmentation

In order to implement continuous mapping, EMG signals were segmented into 200 ms analysis windows. All the processing procedures including motion detection, feature extraction, and classification were performed on each analysis window. In our program, the processing is invoked every 100 ms so the classification result updates every 100 ms based on the latest 200 ms of EMG. It takes approximate 15 ms to process the data (from raw data to classification results, including motion detection, feature calculation, and classification), and the lag between the user's motion and cursor action is <230 ms including the lag of data transfer.

#### Motion Detection

Motion detection aims to determine whether an analysis window contains inactive muscle (i.e., baseline EMG) or active muscle. An analysis window is considered to contain EMG signals from user's movements when the mean absolute value (MAV) of the analysis window is greater than a given threshold, and vice versa. The MAV is calculated as Equation 1, where *EMG*(*c, w*) is the *w*^*th*^ (from 1 to *W*, the duration of the analysis window) data in the *c*^*th*^ (from 1 to *C*, the total number of channels) channel, with offset removed.

(1) $$\begin{array}{r} MAV=\frac{\sum_{c=1}^{C}\sum_{w=1}^{W}|EMG(c,w)|}{C\times W} \end{array}$$

The threshold is set to 3 times the MAV when the user is totally relaxed. If the analysis window does not contain EMG above threshold, the data processing terminates, and the classification result is “no movement.” Otherwise, myoelectric pattern recognition is applied to process the data.

#### EMG Features and Myoelectric Pattern Recognition

For each analysis window of each movement pattern (*Mi, i* = 1, 2, …, 5, listed in Table 1 but excluding “no movement”), the root mean square (RMS) amplitude (24, 25) and the 4th-order autoregressive (AR) model coefficients (18) of each channel were calculated as features. One of the AR model coefficients that is constant was removed, so that there was *F* = 5 features from each channel each analysis window. Features from all channels (a total of *C* = 4 channels) were then concatenated as a feature vector with 1 row and *F* × *C* = 20 columns, denoted as ${\text{S}}_{Mi}^{1,F\times C}$. A naive Gaussian Bayesian classifier (18) with equal-prior-probability assumption was applied to classify the different movement patterns. In our study, the classifier was trained using *N*_*Mi*_ analysis windows of each movement pattern *Mi* before being used for classification, aiming to set up a statistical model for each pattern in the feature space. Specifically, feature vectors of each movement pattern *Mi* formed a training dataset ${\text{S}}_{Mi}^{N_{Mi},F\times C}$ in the form of a matrix with *N*_*Mi*_ rows and *F* × *C* columns. The mean value of each training dataset was used as an estimation of the center of each movement pattern (**CM**_*Mi*_) in the feature space, and the covariance was used as an estimation of the distribution (**DM**_*Mi*_).

Classification aims to determine the movement pattern (*Mx*) of a new analysis window with a feature vector ${\text{S}}_{Mx}^{1,F\times C}$ based on its distance to each movement pattern. With the assumption that the priori probability of each movement pattern was equal, the distance *D*(*Mx, Mi*) was defined as Equation 2. The nearest movement pattern was the classification output. Therefore, the classification result was one of the movements listed in Table 1 (excluding “no movement”).

(2) $$\begin{array}{l} \text{ln}\left(D\left(Mx,Mi\right)\right)=-\frac{1}{2}\text{ln}\left|\text{D}{\text{M}}_{Mi}\right|-\frac{1}{2}\left({\text{S}}_{Mx}^{1,F\times C}-\text{C}{\text{M}}_{Mi}\right)\\ \;{\left(\text{D}{\text{M}}_{Mi}\right)}^{-1}{\left({\text{S}}_{Mx}^{1,F\times C}-\text{C}{\text{M}}_{Mi}\right)}^{T} \end{array}$$

### Cursor Control

The FMMI controls the cursor by sending cursor control commands using the Windows Programming Interface. At the same time, the cursor can be controlled by a regular mouse. It is therefore possible that the user can use a regular mouse for general control, and the interface for precise control.

Each classification result was mapped to a cursor action that was updated every 100 ms. Two types of cursor control commands were used in our customized program running on Windows, i.e., cursor movement commands and left button commands. Each cursor movement command moved the cursor only a few pixels away from its original position, in order to make the cursor movements continuous and smooth. The amount of pixels was configured by the user, which determined the speed and pointing resolution of cursor movement. For example, the default setting (3 pixels per command) meant that the cursor moved at a speed of 30 pixels per second (pps) with a pointing resolution of 3 pixels. Left button commands include the Press command and the Release command, which were triggered only once when the user's bite-down motion began and ended, respectively. If a bite-down motion lasted for more than 1.5 s, no Release command was sent, so that the user could perform other facial movements while Windows performed as the button was being pressed. In this case, a “ding” sound occurred when the bite-down motion reached 1.5 s, in order to notify the user that the button was pressed and held. A different “ding” sound occurred when the button was released later.

### Subjects

Ten able-bodied subjects (S1–S10, 37.7 ± 11.5 years old, 7 males and 3 females) participated. All subjects gave written informed consent before any experimental procedures. The experimental protocols were approved by the Committee for the Protection of Human Subjects (CPHS) of the University of Texas Health Science Center at Houston and TIRR Memorial Hermann Hospital (Houston, TX, USA).

### Experimental Protocol

**Figure 3:**
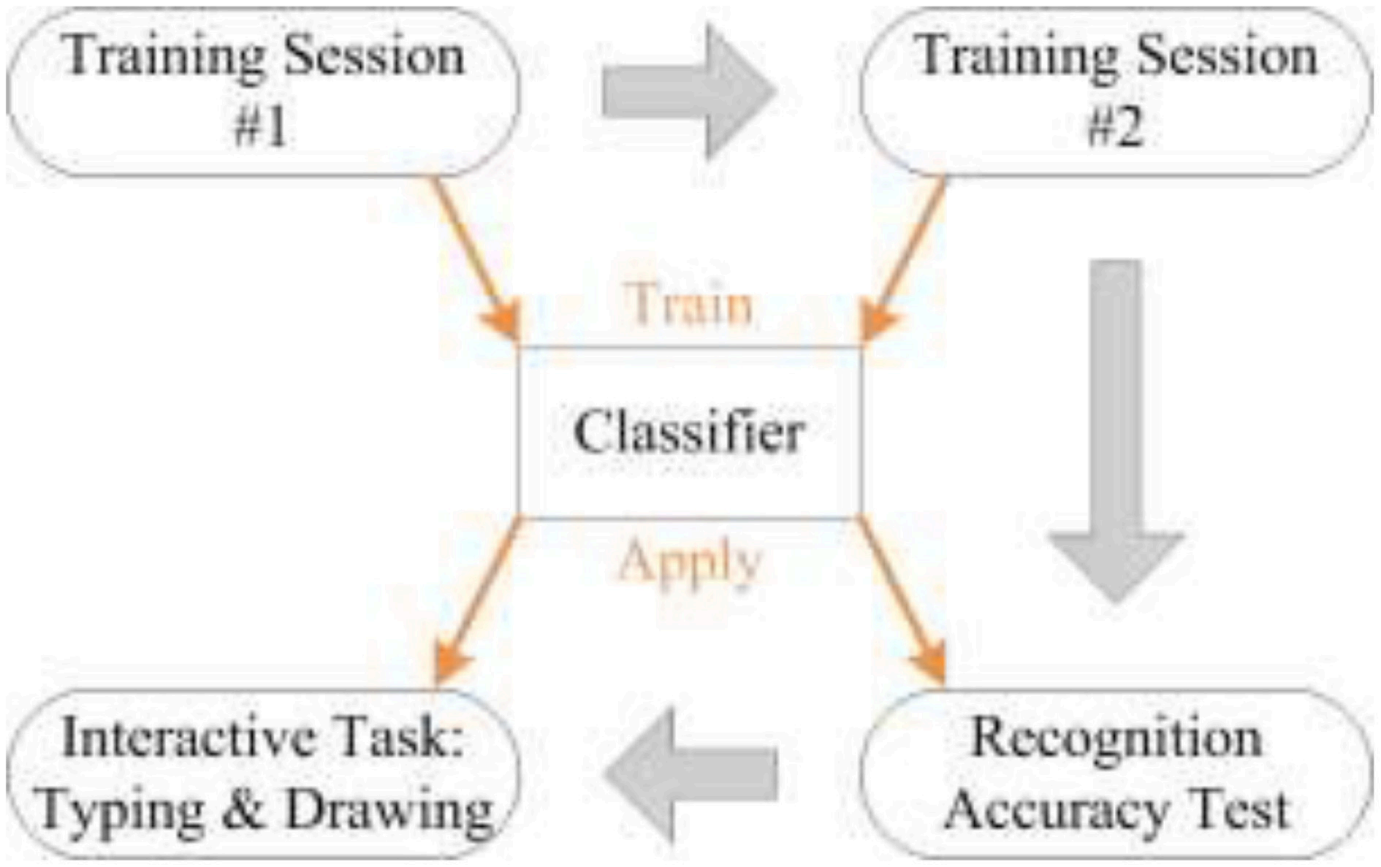
Cascade of experimental procedures.

Each subject made one visit to the laboratory. The experiment lasted about 2 h and consisted of four sessions (Figure 3). Before the first session, each subject learned all the facial movements as well as the mapping, and was given time to practice these facial movements. The fourth session included two interactive tasks that were randomly assigned. In each experiment, the subject was seated comfortably on a chair and faced a computer screen for real-time feedback. The cursor movement was set to 3 pixels per command in all sessions.

A classifier was trained for each subject. In the first and second sessions, EMG signals were recorded in order to train the classifier. The classifier was trained using these data before the third session, and then its classification accuracy was measured by experiments in the third session. In the fourth session, the subject was given two interactive tasks in order to measure the FMMI performance.

#### Training Sessions #1 and #2

In the first session, the subject performed each facial movement 10 times, each lasting about 2 s, with a 2-second rest after each motion. The subject repeated the same procedure in the second session. Therefore, approximately 40 s of EMG for each facial movement was recorded from these two sessions, and were used to train a naive classifier. Each session lasted about 5 min, and there was a 2-min rest between the two sessions. The cursor was not controlled by the subject in the first two sessions. However, real-time visual feedback was available (including a red picture box that appears when a facial movement is detected and being recorded, as well as a chart showing the MAV calculated in real time based on Equation 1 and the threshold), so that the subject could know if his movement had been accepted, and could also get a better understanding about the threshold of motion detection.

#### Recognition Accuracy Test

The recognition accuracy test was conducted in the third session, in which the subject's facial movements were classified in real time by the classifier trained using data recorded in the first two sessions (about 400 analysis windows for each pattern). The subject repeated each facial movement a few times until 100 classification results were obtained for each pattern (excluding “no movement”). The recognition accuracy was then calculated as the ratio of the number of correct classification results to total classification results (e.g., accuracy of “left side smirk”= the number of “left side smirk” that is correctly classified/100).

In order to compare pattern-based mapping and channel-based mapping, EMG data recorded in this session were processed using channel-based mapping after the experiment. Similar to (1) and (2), RMS of each channel was first calibrated, and then the channel with the maximal RMS was picked out to represent the motion. For example, a motion will be mapped to “Moving up” when the leading RMS value comes from Mentalis. The calibration parameter of each muscle are the mean RMS value of that muscle in the first two sessions when the muscle is the only activated muscle. The pattern “Moving down” was excluded because only four patterns can be mapped to by using four channels.

#### Interactive Tasks

In order to measure the interaction performance of the FMMI, two interactive tasks were conducted in the fourth session. In this session, the subject had full control of the system cursor, which was mapped from the classification results. Before performing the tasks, the subject was given sufficient time to practice, in order to get familiar with the facial movements, the mapping, and our system. The subject needed to get used to misclassifications (i.e., the subject's movement is classified incorrectly so that the cursor's action is different from the subject's expectation). Besides, the subject learned how to use Windows On-Screen Keyboard and Windows Paint, the applications that were used in this session. For each task, cursor actions (including trace) and time to accomplish the task were recorded by our program. The clock started when the subject first move the cursor, and stopped by the experimenter when the task was done. The time between the first and the last cursor action was calculated by the program as the time to accomplish the task.

##### Typing task

In the typing task, the subject was asked to type a sentence “I'm using facial control.” in a textbox using Windows On-Screen Keyboard. The subject was allowed to use any way to type (e.g., use either the “Caps Lock” or “Shift” key to input the letter “I”), and had to correct any error occurred during typing. The task began with the cursor placed at the top left of the screen keyboard, and ended when the typing was done. Each subject had up to three chances to accomplish the task.

Path efficiency (PE) (2) was calculated based on the cursor trace in the typing task. It was defined as the ratio of the length of the optimal path to the length of the subject's trace. The difference between the lengths of the two paths is usually caused by incorrect classification results or the subject's mistakes. Therefore, a higher path efficiency usually represents a better interaction performance. In our design, the cursor can only move in four directions. Manhattan Distance (MD) was therefore applied to measure the length of the optimal path. Given the position *Pos*_*i*_ = (*x*_*i*_, *y*_*i*_) where the user correctly clicked the i^th^ screen key, the length of the optimal path from the previous screen key to the i^th^ screen key can be calculated using Equation 3.

(3) $$\begin{array}{r} MD\left(Pos_{i-1},Pos_{i}\right)=|x_{i}-x_{i-1}|+|y_{i}-y_{i-1}| \end{array}$$

Thus, path efficiency can be calculated using Equation 4, where the length of the subject's trace (LS) is the sum of each cursor movement.

(4) $$\begin{array}{r} PE=\frac{\sum_{i}MD(Pos_{i-1},Pos_{i})}{LS} \end{array}$$

According to Fitts's law (9), the time required to move the cursor from one screen key to the target screen key is a function of the task's Index of Difficulty (ID). ID can be estimated using the distance and the width of the target screen key (9). In order to quantify the relation between movement time and ID, four horizontal movements (e.g., from “f” to “a” on the screen keyboard) were picked out as samples to perform a linear fitting.

##### Drawing task

In the drawing task, the subject was asked to draw a simplified mushroom (a semi-circle on the top plus a rectangle on the bottom) using Windows Paint. A sample picture was provided to the subject. The subject was allowed to draw it at any place on the canvas using any tool available in Windows Paint (e.g., straight line, curve, and rectangle). The task began with the cursor placed at the top left of the Windows Paint, and ended when the drawing was done. Each subject had up to three chances to accomplish the task.

## Results

### Cursor Control Accuracy

Table 2 shows each subject's facial movement classification accuracy obtained in the third session, along with the standard deviation of the five movement patterns. All subjects achieved high accuracies (96.8 ± 0.9% with the median at 98.0%). Six subjects achieved very high accuracies (above 97%), and the lowest accuracy was 91.3%. Subject S8 had the largest standard deviation because of a low accuracy during the “right side smirk” movement. Table 3 shows the average confusion table with the standard deviation among all the subjects when using pattern-based mapping. All the 5 elements on the diagonal, which correspond to the percent of correct classification, are >93%, and their standard deviations are all below 10%. Thirteen out of the 20 off-diagonal elements, which correspond to misclassification, are below 1%. Table 4 shows the average confusion table with the standard deviation when using channel-based mapping. All the 4 elements on the diagonal are smaller than 93%, and their standard deviations are all above 10%. Three out of the 12 off-diagonal elements are below 1%. Non-negligible individual differences in accuracy were observed when using channel-based mapping. The average accuracy across all subjects was 88.2 ± 4.7%. Only six subjects achieved an accuracy about the same level when using pattern-based mapping (i.e., above 90%), and the lowest accuracy was 51.2%.

**Table 2:** Facial movement classification accuracy of each subject.

Subject	S1	S2	S3	S4	S5
Accuracy (%)	98.2 ± 1.7	91.3 ± 7.0	98.2 ± 3.6	100.0 ± 0.0	94.0 ± 6.8
Subject	S6	S7	S8	S9	S10
Accuracy (%)	99.0 ± 1.1	95.4 ± 6.7	94.1 ± 13.3	97.8 ± 2.7	100.0 ± 0.0

**Table 3:** Confusion table of facial movement classification.

**Facial movement**	**Classification output (%)**				
	**Raise lower lip**	**Close lips**	**Left side smirk**	**Right side smirk**	**Bite down**
Raise lower lip	97.7 ± 5.7	1.9 ± 5.0	0.2 ± 0.6	0.2 ± 0.6	0.0 ± 0.0
Close lips	2.1 ± 3.8	95.5 ± 5.9	1.1 ± 2.4	1.1 ± 2.6	0.2 ± 0.6
Left side smirk	0.2 ± 0.6	1.2 ± 2.0	98.0 ± 2.2	0.5 ± 1.6	0.1 ± 0.3
Right side smirk	1.8 ± 3.9	4.0 ± 8.0	0.1 ± 0.3	93.8 ± 9.6	0.3 ± 0.9
Bite down	0.1 ± 0.3	0.1 ± 0.3	0.1 ± 0.3	0.0 ± 0.0	99.7 ± 0.7

**Table 4:** Confusion table of channel-based facial movement mapping.

**Facial movement**	**Mapping output (%)**				
	**Raise lower lip**	**Close lips**	**Left side smirk**	**Right side smirk**	**Bite down**
Raise lower lip	89.3 ± 15.0	–	4.3 ± 6.4	5.1 ± 9.9	1.3 ± 4.1
Close lips	–	–	–	–	–
Left side smirk	3.0 ± 5.1	–	88.8 ± 18.7	8.0 ± 19.1	0.2 ± 0.6
Right side smirk	6.8 ± 13.9	–	9.6 ± 27.9	83.4 ± 29.4	0.2 ± 0.6
Bite down	0.8 ± 1.5	–	1.5 ± 4.4	6.1 ± 13.5	91.6 ± 14.0

### Typing Task Performance

**Figure 4:**
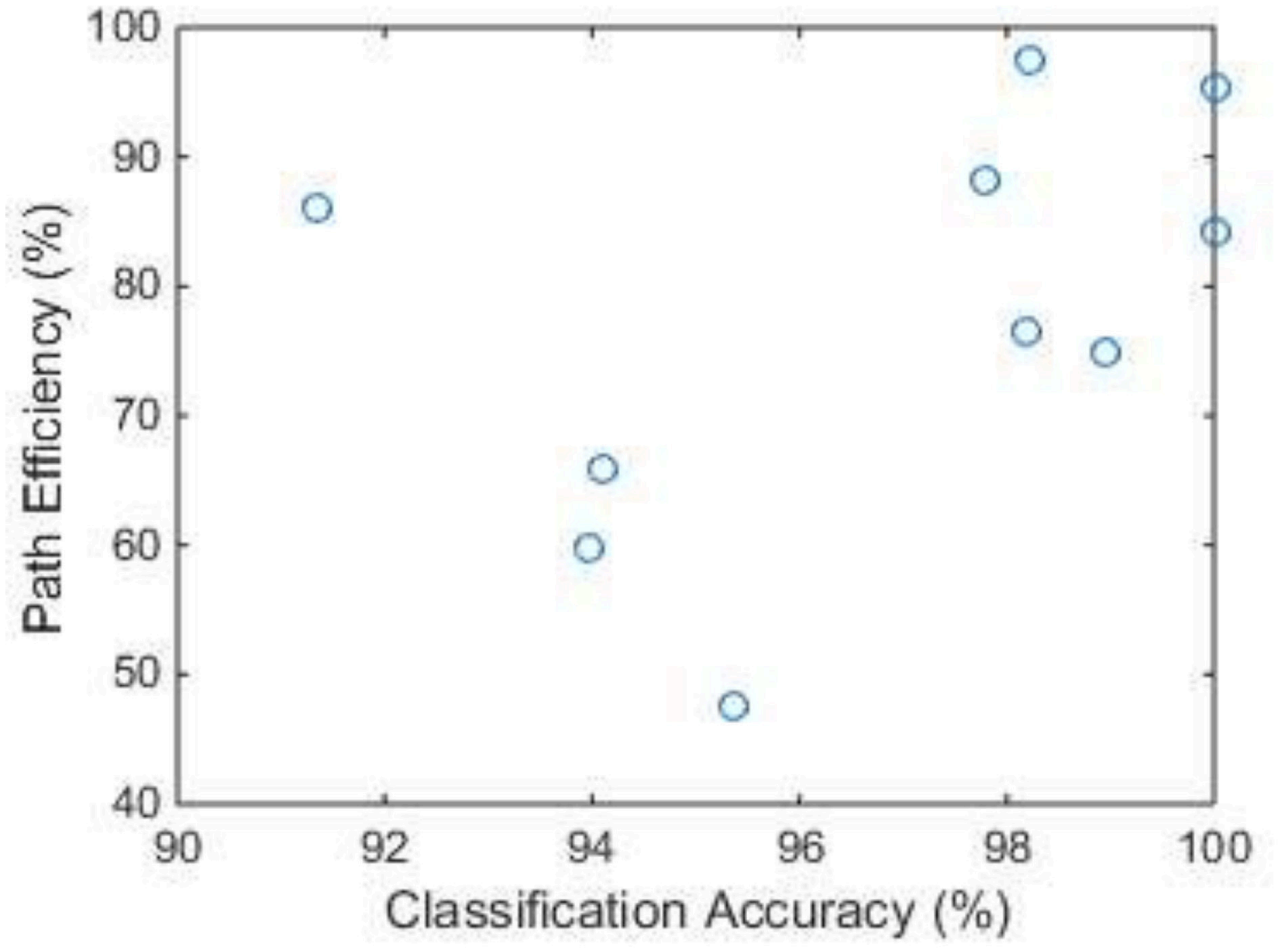
Path efficiency in the typing task of each subject. The x axis is the accuracy of classification results obtained from the third session.

Figure 4 shows the calculated path efficiency of each subject in the typing task. The median path efficiency was 80.4% (with quantiles 65.8 and 88.3%). Subjects with higher classification accuracies (>97%) tended to achieve greater path efficiency, but no significant correlation between classification accuracies and path efficiency was observed (Pearson correlation coefficient *r* = 0.447, *p* = 0.195). Seven subjects achieved high path efficiencies (75% and higher), six of whom achieved high classification accuracies in the third session. The only exception was the youngest subject (27 years), who achieved a high path efficiency with a relatively low accuracy. Interestingly, the subject with the lowest path efficiency was the oldest (65 years), who had more mistakes when performing the task.

**Figure 5:**
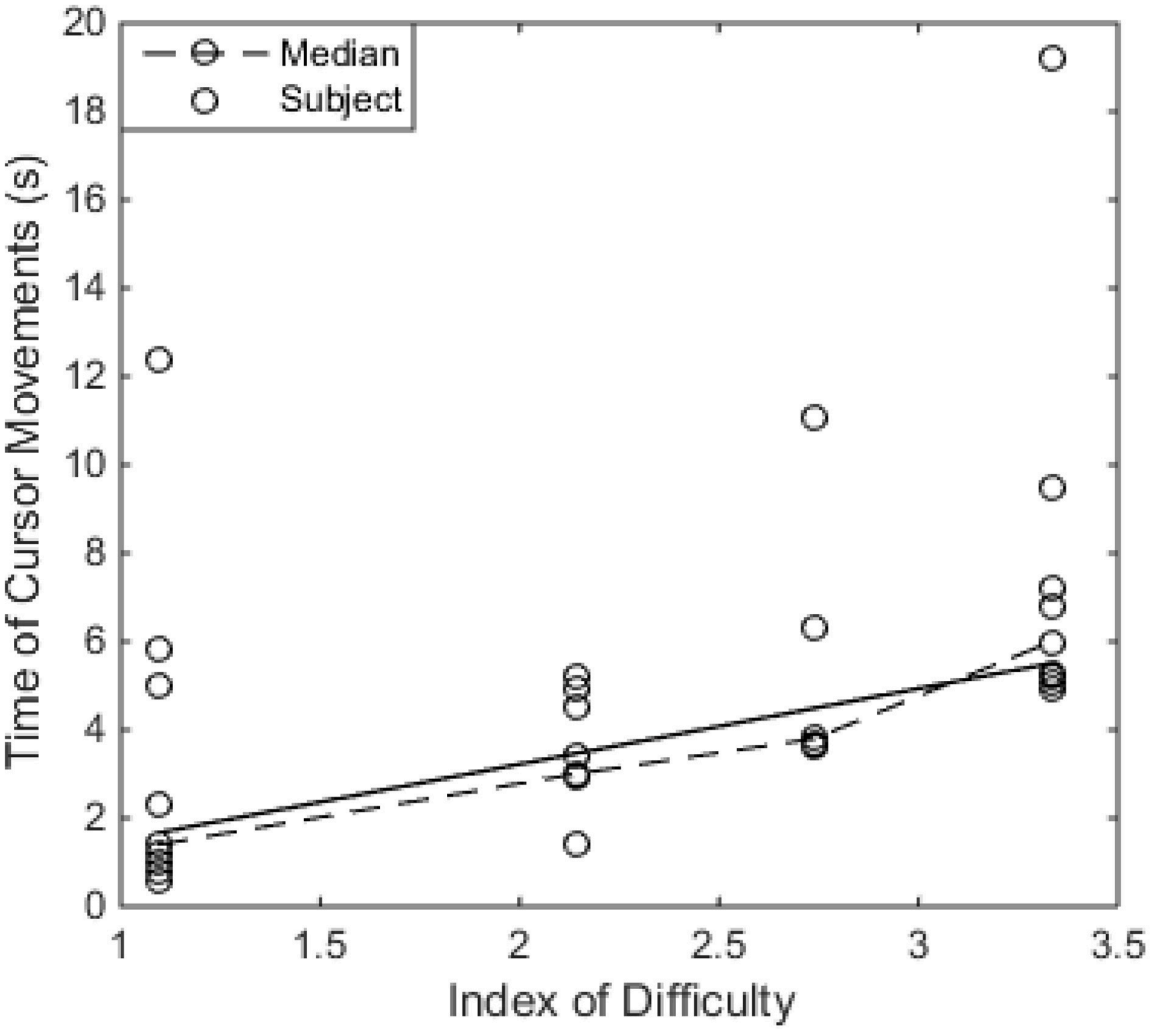
The relation between Time of Cursor Movements and Index of Difficulty (ID) in the typing task. The dash line shows the median value at each ID level, and the solid line shows the linear fitting result of the median values.

Figure 5 shows the relation between time and ID in the typing task, which are defined in Fitts's Law. The time was measured from the first cursor movement after the previous click to the last cursor movement before another click. At each ID level, the time needed to move the cursor appeared to be similar among different subjects, except a few outliers (i.e., did not follow a normal distribution). Therefore, the median value at each ID level was used to represent all subjects. A linear relation (also a significant correlation with *r* = 0.962, *p* = 0.038) between the median time and ID level was found as Equation 5.

(5) $$\begin{array}{r} Time=-0.2044+1.7135\times ID \end{array}$$

**Figure 6:**
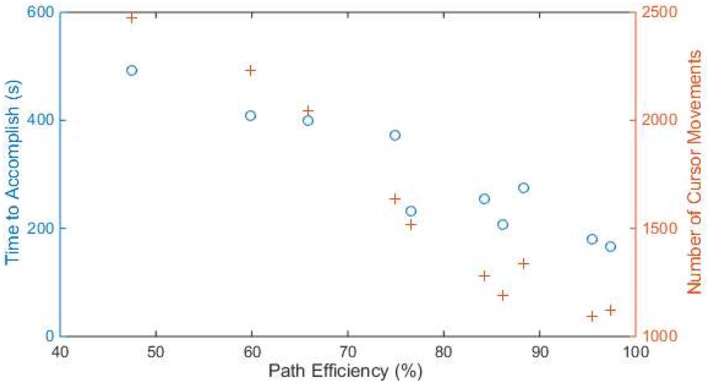
The time (blue circle) and number of cursor movements (orange cross) of each subject to accomplish the typing task. The x axis is the path efficiency in the typing task. Samples with the same x-value are from the same subject.

Figure 6 shows the total time and number of cursor movements that each subject used to accomplish the typing task. The median time was 265.4 s (about 5.9 letters per minute, with quantiles 206.3 s and 400.5 s), and the median number of movements was 1,425 (with quantiles 1,192 and 2,044). It appears that subjects with higher path efficiency used less time (*r* = −0.942, *p* = 0.000) and fewer movements (*r* = −0.980, *p* = 0.000) to accomplish the typing task.

### Drawing Task Performance

**Figure 7:**
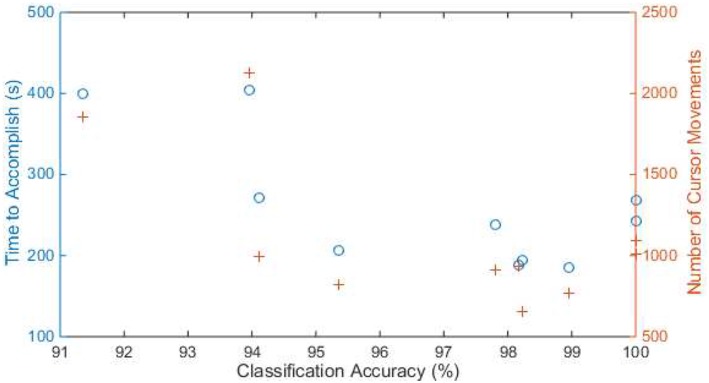
The time (blue circle) and number of cursor movements (orange cross) of each subject to accomplish the drawing task. The x axis is the accuracy of classification results obtained from the third session. Samples with the same x-value are from the same subject.

**Figure 8:**
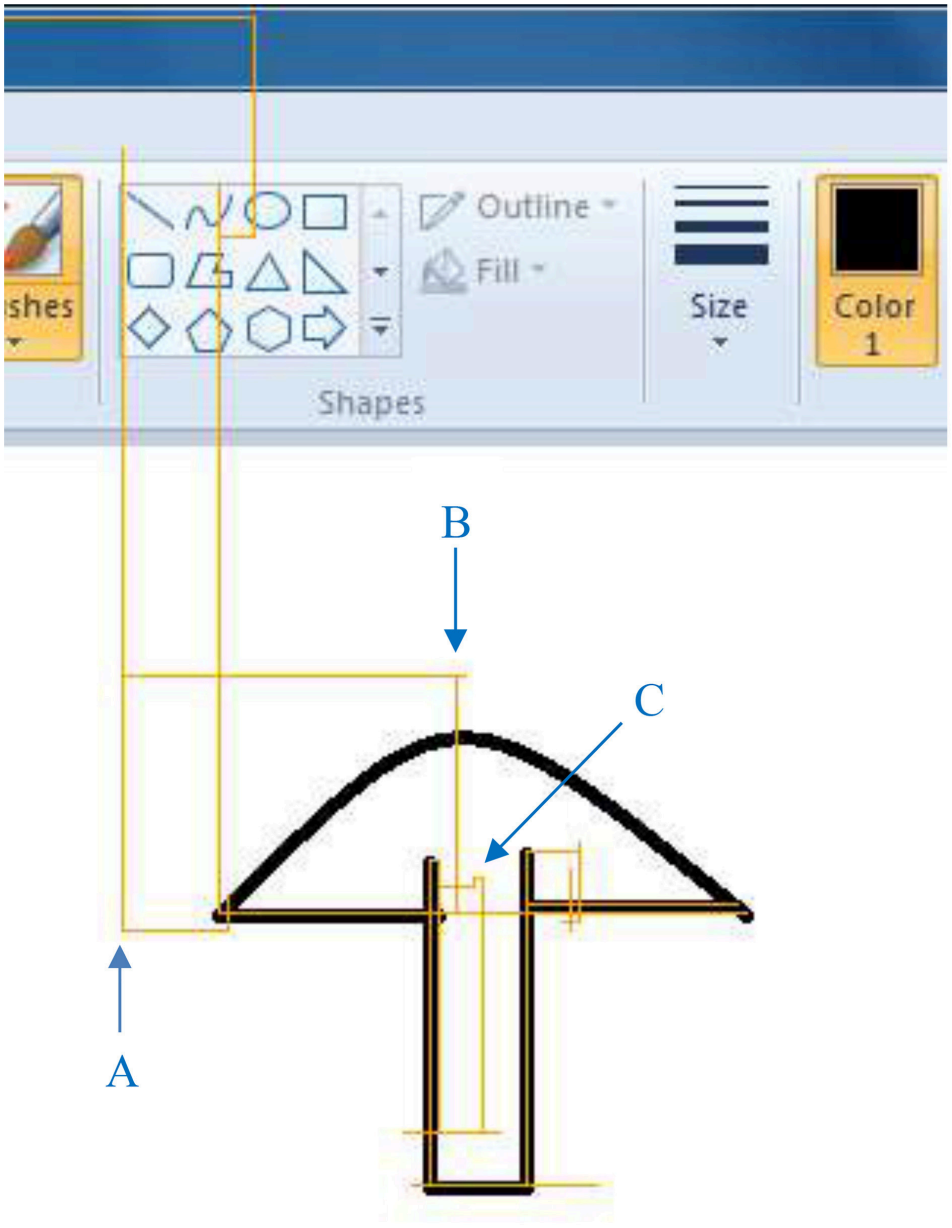
An example of the drawing (black) with the subject's cursor trace (orange). Examples of correct motion transition **(A)**, overshooting **(B)**, and misclassification **(C)** are marked in blue.

Figure 7 shows the total time and number of cursor movements that each subject used to accomplish the drawing task. The median time was 239.9 s (with quantiles 194.2 and 271.9 s), and the median number of movements was 966 (with quantiles 819 and 1,091). Subjects with higher classification accuracy spent less time (*r* = −0.716, *p* = 0.020) and fewer movements (*r* = −0.663, *p* = 0.036) to accomplish the drawing task. Figure 8 is an example of a subject's drawing with the cursor trace.

## Discussion

In this study, we demonstrated the performance of human-computer interactions using a cursor controlled by facial movements. Different from conventional mapping schemes, facial movements were detected and classified using myoelectric pattern recognition algorithms, and mapped to cursor actions in our design. By using such a mapping scheme, only three EMG channels were needed to control the cursor movement in four directions, compared with recent studies in which four EMG channels were necessary (2, 3, 9). By using only four EMG channels in our design, full control of a cursor (including movement in four directions, click, and drag & drop) was implemented. Furthermore, the algorithms were able to learn the EMG patterns of each subject, so that there was no need to calibrate or tune those channels manually. Although it is possible to map these four channels to four out of five movement patterns using a channel-based mapping and manually determined thresholds, its mapping accuracy was lower than that of pattern-based mapping. Only a portion of subjects (60% in our experiments) achieved a relatively high accuracy. Those subjects who achieved relatively low accuracies would have many incorrect mappings in interaction tasks, which bring down their interaction experience. Besides, interaction task cannot be accomplished using only four patterns (i.e., without “move down”). In other words, an additional EMG channel is necessary for interaction tasks if using channel-based mapping. The subject with the lowest channel-based mapping accuracy had non-negligible co-activation among three out of four channels, which is a challenge for channel-based mapping but not for pattern-based mapping. In our pattern-based experiments, all subjects achieved high accuracies, half of them achieved real-time classification accuracies above 98%, which is very high for real-time classification. Among the five movement patterns, the “Bite down” movement has the best classification performance, because it is performed by a separate muscle, compared with the other four mouth movements, which are performed by three small muscles around the mouth. Besides, the three muscles change shapes when performing mouth movements, which affects the contact between our electrodes and the skin, introducing noise and artifacts. However, these four mouth movements also achieved good classification movements with accuracies between 93.8 and 98.0%, indicating that the myoelectric technique was accurate and reliable. Path efficiency is an indicator that reflects the quality of interaction. High path efficiency can only be achieved if the interaction system is accurate (e.g., low misclassification rate) and easy to use (e.g., minimal overshooting). The path efficiencies in our experiment were higher than a recent study on facial movement-based cursor control systems, in which subjects' path efficiencies ranged from 60 to 80% (2). Although we applied a different mapping scheme, it is still feasible to compare path efficiency between our system and existing systems. In (2) for example, the shortest path from one position to another is the Euclidean distance, so that their calculation is based on Euclidean distance. However, the shortest path in our system is the Manhattan distance. Thus, our path efficiency definition is based on Manhattan distance. As a result, both systems have the same upper limit of 100% for path efficiency, and the calculation will not be affected by the position of start or end point of the path. Therefore, the layout of the typing board does not necessarily change the path efficiency, although it may change the interaction efficiency.

The speed of cursor movement is usually controlled by the amplitude of the user's EMG signals when conventional channel-based mapping scheme is applied (2, 9). In our design, the speed was constant, so that the user can pay more attention to the movement pattern. Considering that the user has full control of the system cursor, he or she can change the speed on our application's graphic interface at any time. Hence, the user can use the FMMI in a variety of cases, from large-scale movements to precise operations. It is also believed that speed affects interaction performance. For example, using high speed saves time of cursor movement, but increases the chance of overshooting. Therefore, it is meaningless to compare interaction performance of a channel-based mapping (EMG controlled speed) with our system (constant speed). On the other hand, high mapping accuracy or classification accuracy tends to provide better interaction performance as shown in Figure 7. Therefore, we believe that high interaction performance cannot be achieved by using channel-based mapping on some subjects in our experiments because of their relatively low accuracies. However, it does not mean channel-based mapping is outperformed by pattern-based mapping. Interface using channel-based mapping can provide high input rate in some use case [e.g., moving left and up at the same time (2)], while pattern-based mapping provides a more flexible solution (e.g., fewer EMG channels are required, and the algorithms can learn customized patterns) and performed better in our use settings.

It is very common that the user has to use customized tools for interactions when using a FMMI. For example, users used a customized input panel for typing in previous studies (2) and (3). Such customized tools could have significantly increased the interaction efficiency, so that the input speed of the typing task (i.e., the number of letters per minute) in their experiments was much higher than that in the present study, though we had higher path efficiency. However, it is impractical to develop a tool or an input panel for every interaction task. Therefore, our design, which is taking over the system cursor, gives the user a universal interface to manipulate the computer. Furthermore, the user's experience of using a regular mouse can be applied when using our interface. It can accelerate the learning process, and more importantly, it can make some operations much more efficient. For example, if the user wants to delete a whole paragraph when typing, he / she can easily select the text and click the delete key on the screen keyboard when using our interface. However, the user may have to delete one letter at a time when using the aforementioned input panels.

As a pattern-based control scheme, only one pattern is accepted at a time. As a result, the cursor can only move in one of four directions, as shown in Figure 8. It sometimes makes the path longer, but it is good in some cases as well (e.g., drawing a horizontal line). It is noteworthy that almost all the computer operations can be done with movements in only four directions, including drawing a curve as in our experiment. Moreover, movement transitions may affect interaction performance because they are usually non-stationary or in ambiguous states. Specifically, there are two types of movement transitions, (1) transitions between a motion and the rest state (i.e., the beginning or end of a motion), and (2) transitions between two motions. Our motion detection algorithm is designed to be able to filter out the beginning or end of a motion based on two of our observations. First, the beginning or end of a motion are usually small in the EMG amplitude. Secondly, an analysis window that contains the beginning or end of a movement usually contains a baseline before or after the movement as well. Therefore, the MAV of an analysis window that contains either beginning or end of a motion is usually too small to reach the threshold of motion detection. Transitions between two motions can happen in the case of overshooting and making turns, and misclassifications caused by transitions can be captured by the cursor trace such as Figure 8. For example, a misclassification when making a turn can introduce drift in other directions and usually makes jitters in the trace as shown in Figure 8 (marked as “C”). However, this is the only misclassification that happened in transitions in this experiment, and it did not happen often in other experiments. We noticed that in most cases the subject moved the cursor in one direction, and then made a stop before moving in another direction, although the subject was never commanded to make a stop between two motions. As a result, movement transitions did not significantly affect the performance of our system. However, all these results and observations were under laboratory conditions. In daily use, a voting strategy over two or more analysis windows that only accepts the classification with the highest votes e.g., (26) can be applied if movement transitions affect the user's experience. It can filter out occasional misclassifications as well. Additional filters may be needed in real life settings to preprocess EMG dada contain noise or artifacts.

Cler et al. (2) discovered that both discrete and continuous mapping can achieve high performance. Typical pattern-based discrete mapping generates one control command when one motion is detected (13, 28), thus the update rate of control commands is usually below 1 Hz. If the cursor moves 3 pixels each time, more than 300 motions are required in order to move the cursor from far left to far right, which may take several minutes. In contrast, control command can be updated at 10 Hz or higher when using continuous mapping (29). Therefore, continuous mapping with an update rate at 10 Hz was applied in our design.

Although high path efficiencies can be achieved when compared with other facial movement-machine interaction systems, there is still a large gap between our system and a regular mouse. Our system has a low input rate, so that the user needs a long time to accomplish the tasks. Our future work involves developing advanced ways allowing the user to control the cursor movement speed. Patients with limited hand function will be recruited to assess the performance of the system.

## Conclusion

A facial movement-machine interface was developed in this study in order to help users with limited hand function manipulate electronic devices. Facial movements were detected using four EMG sensors, and five movement patterns were classified using myoelectric pattern recognition algorithms. The results from 10 able-bodied subjects show that facial movements can be detected and classified at high accuracies. The pattern-based continuous mapping between facial movements and cursor actions achieved high performance in both a typing task and a drawing task.

## Ethics Statement

This study was approved by the Committee for the Protection of Human Subjects (CPHS) of The University of Texas Health Science Center at Houston and TIRR Memorial Hermann (Houston, TX, USA). All procedures of the study were performed in accordance with the Declaration of Helsinki. The subject gave written and informed consent before the experimental procedures.

## Author Contributions

ZL experimental design, subject recruitment, data collection, data analysis, and first draft of the manuscript. PZ conception, study design, data analysis, supervision, and manuscript revision. Both authors approved the final manuscript.

### Conflict of Interest Statement

The authors declare that the research was conducted in the absence of any commercial or financial relationships that could be construed as a potential conflict of interest.
